# Effects of Non-Suspended Robot-Assisted Ambulatory Training on Stroke Patients

**DOI:** 10.3390/healthcare14131990

**Published:** 2026-07-03

**Authors:** Wen-Fang Lei, Shin-Da Lee

**Affiliations:** 1 PhD Program for Health Science and Industry, China Medical University, Taichung 406040, Taiwan; vanessalei5898@gmail.com; 2 PhD Program in Healthcare Science, China Medical University, Taichung 406040, Taiwan

**Keywords:** CVA, hemiplegia, gait training, robot-assisted therapy, walking ability

## Abstract

**Background:** The study aimed to investigate the effects of non-suspended robot-assisted ambulatory training with approximately 80–100% weight-bearing during the stance phase on one leg and 0% weight-bearing support during the swing phase on the other leg on hemiplegic stroke patients who were unable to ambulate at baseline. Traditional robot-assisted gait training commonly provided substantial body-weight support (approximately 0–20% weight-bearing during the stance phase on one leg), whereas the present system enables near-normal weight-bearing during gait training. **Methods:** Pre- and post-assessments of Brunnstrom stage, standing balance, and the Barthel Index of Activities of Daily Living (ADL) were performed in sixty hemiplegic stroke patients (30 right-sided and 30 left-sided hemiplegia) with stroke onset less than 6 months and without ambulatory ability. A retrospective controlled study was performed using a non-suspended robot-assisted ambulatory training machine (RAATM) that provides approximately 80–100% weight-bearing during the stance phase on one leg for more than 150 min a month (>4500 guided steps), combined with a 4-week conventional rehabilitation program (RAATM group, *n* = 30). Outcomes were compared with those of an age-, affected side-, and baseline walking-ability-matched control group (control group, *n* = 30) that received only a 4-week conventional rehabilitation program. **Results:** The average accumulated intervention duration in the RAATM group was 246 ± 74 min, which received intervention of RAATM with 80–100% weight-bearing during the stance phase on one leg and 0% weight-bearing during the swing phase on the other leg. The pre-to-post changes in the Brunnstrom stage of the lower extremities, static standing balance score, dynamic standing balance score, mobility on level surfaces, stairs, and total Barthel Index score were significantly higher in the RAATM group than in the control group. **Conclusions:** Functions of lower extremities, standing balance, and mobility ability can be improved after intervention of non-suspended RAATM within a month. Non-suspended robot-assisted ambulatory training appeared to be an effective therapeutic approach for hemiplegic stroke patients pre-assessed without ambulatory ability.

## 1. Introduction

Stroke remains the second leading cause of death and one of the leading causes of long-term neurological disability worldwide [1]. Impaired walking ability is among the most common consequences of stroke and substantially limits independent mobility, community participation, and activities of daily living (ADL) [2]. Approximately 65% of stroke survivors exhibit reduced ambulatory capacity [3], and nearly 20% remain unable to walk independently one year after stroke onset [4]. Because the greatest neurological recovery generally occurs during the early post-stroke period, particularly within the first three months [5], rehabilitation strategies that facilitate early restoration of walking ability are critical for maximizing functional recovery.

Conventional gait rehabilitation programs rely heavily on therapist-assisted overground walking, treadmill training, and task-oriented functional exercises.

Although these approaches can improve walking performance, their effectiveness may be limited in patients with severe motor impairment who require substantial physical assistance and intensive supervision. In addition, concerns regarding patient safety, therapist workload, and training intensity may restrict the amount of repetitive walking practice that can be delivered during rehabilitation [2]. To address these limitations, robot-assisted gait training has increasingly been incorporated into stroke rehabilitation programs [6,7,8,9,10,11,12,13,14,15,16,17,18].

The present study investigated a non-suspended robot-assisted ambulatory training machine (RAATM) designed to provide repetitive gait training under near-normal physiological loading conditions. Unlike conventional suspension-based robotic systems, the device enables approximately 80–100% physiological weight-bearing during the stance phase while mechanically guiding the swing phase through a closed-kinetic-chain mechanism. Such a design may better replicate real-world walking conditions and promote motor recovery through repetitive physiological weight transfer and proprioceptive stimulation.

Current evidence indicates that robot-assisted gait training after stroke has become an important component of contemporary stroke rehabilitation because it enables repetitive, task-specific, and intensive gait practice that may facilitate motor relearning and neuroplasticity [6,7,8,9,10,11,12,13,14,15,16,17,18]. Various robotic systems have been developed, including treadmill-based exoskeletons, end-effector devices, wearable robotic orthoses, ankle rehabilitation robots, and hybrid assistive limb technologies [6,7,8,9,10,11,12,13,14,15,16,17,18].

Numerous clinical studies have demonstrated the beneficial effects of robot-assisted rehabilitation on walking ability, balance control, lower-extremity motor function, and functional independence after stroke [9,12,13,17,18]. Moreover, recent systematic reviews and meta-analyses continue to support the clinical value of robotic rehabilitation. Mehrholz et al. reported that electromechanical-assisted gait training combined with physiotherapy increased the likelihood of regaining independent walking after stroke [6]. Similarly, Lin et al. demonstrated significant improvements in motor function following hybrid robot-assisted gait training in subacute stroke patients [19]. Most recently, Hao et al. concluded that lower-limb rehabilitation robots significantly improve lower-extremity motor function and mobility outcomes in stroke populations [20].

Collectively, current evidence indicates that robot-assisted gait training remains an active area of clinical and scientific investigation, with continuing efforts directed toward optimizing rehabilitation protocols and improving functional outcomes after stroke.

### Limitations of Existing Robotic Gait-Training Systems

Despite encouraging outcomes, most commercially available robotic gait-training systems rely on body-weight support mechanisms that partially unload the lower extremities during gait practice [7,10,12]. Suspension-based systems improve safety and facilitate early mobilization; however, reduced physiological loading may alter sensory feedback, limit symmetrical weight transfer, and decrease task-specific weight-bearing during walking practice.

The factor of weight-bearing remarkably influences motor learning processes that are important for gait recovery after stroke [21]. Furthermore, many previous studies have focused primarily on outcomes such as walking speed, gait endurance, and functional ambulation, whereas relatively few investigations have specifically examined the influence of physiological weight-bearing during robotic rehabilitation [6,19,22].

Although substantial evidence supports the effectiveness of robot-assisted gait training after stroke, several important knowledge and research gaps remain. Most published studies have evaluated robotic gait training systems by employing partial body-weight support through suspension-based mechanisms [7,10,12]. Clinical evidence regarding robotic ambulatory training performed under near-normal physiological weight-bearing conditions remains limited. Second, relatively few investigations have examined non-suspended robot-assisted ambulatory training capable of providing approximately 80–100% physiological weight-bearing during the stance phase while mechanically guiding the swing phase. Third, limited evidence is available regarding the effectiveness of such non-suspended robotic systems in severely impaired stroke patients who are unable to ambulate independently during the early stages of recovery [19]. Therefore, whether greater physiological loading during robotic ambulatory training contributes to superior motor recovery, balance performance, and functional independence remains incompletely understood.

The objective and contribution of the present study are as follows: To address the knowledge gaps, the present study evaluated a non-suspended robot-assisted ambulatory training machine (RAATM) providing approximately 80–100% physiological weight-bearing during the stance phase and minimal weight-bearing during the swing phase in severely impaired stroke patients who were unable to ambulate independently during the early stages of recovery. Unlike conventional suspension-based robotic gait-training systems, the present device utilizes a six-point support structure and a closed-kinetic-chain design that allows repetitive gait training under near-normal loading conditions. The primary objective of this study was to determine whether the addition of RAATM to conventional rehabilitation would improve lower-extremity motor recovery, standing balance, mobility performance, and activities of daily living in hemiplegic stroke patients who were unable to ambulate independently at baseline.

To evaluate these effects, outcome measures reflecting motor recovery, balance function, and functional independence were selected. Brunnstrom stages were used to assess lower-extremity motor recovery [23,24,25], Balance Assessment scores were used to evaluate postural control and mobility capacity [26], and the Barthel Index was used to assess independence in activities of daily living [27,28]. Standing balance assessments provide important information regarding postural control and mobility capacity [26].

We hypothesized that non-suspended robot-assisted ambulatory training with approximately 80–100% physiological weight-bearing during the stance phase in severely impaired stroke patients who are unable to ambulate independently during the early stages of recovery would produce greater improvements in lower-extremity motor recovery, standing balance, and ADL performance than conventional rehabilitation alone.

## 2. Materials and Methods

### 2.1. Study Design

This study employed a retrospective matched-control pretest–posttest design to investigate the effects of a non-suspended robot-assisted ambulatory training machine (HIWIN Robotic Gait-Training Device, HIWIN Technologies Corp., Taichung, Taiwan, RAATM) in hemiplegic stroke patients without independent ambulatory ability at baseline. Medical records of eligible inpatients admitted between October 2014 and October 2015 were reviewed. Participants who received RAATM in addition to conventional rehabilitation constituted the intervention group, whereas control participants were selected from the same rehabilitation unit and received conventional rehabilitation alone.

To reduce selection bias, control participants were matched to RAATM participants according to age, hemiplegic side (right or left), ambulatory status at baseline, and stroke onset duration. Outcome assessments were performed before and after the 4-week intervention period.

Because of the retrospective matched-control design, random allocation was not performed. Eligible control participants were selected from hospital records by an independent physician who was not involved in outcome assessments or intervention delivery. Outcome assessments were performed by experienced therapists who were blinded to group allocation and study hypotheses. Data coding was completed before statistical analysis, and the statistician remained blinded to group identity until completion of the analyses.

### 2.2. Hemiplegic Stroke Patients

Hemiplegic stroke inpatients were sampled according to inclusion criteria and exclusion criteria in the Department of Rehabilitation Medicine, Chung Shan Medical University Hospital, Taiwan, from 16 October 2014 to 15 October 2015. The inclusion criteria were (1) hemiplegic stroke patients with either right or left hemiplegic side without independently ambulatory ability, (2) stroke for the first time, (3) less than 6 months elapsed after the incident and (4) age between 45 and 75 years. The exclusion criteria were (1) other CNS disorders, including multiple sclerosis, Parkinson’s disease, and head injury, (2) additional psychological or medical conditions deeming a patient unable to participate in this study, (3) ataxia, dyskinesia, or peripheral neural lesions, and (6) a serious cardiovascular disease. The diagnosis of hemiplegic stroke patients was based on clinical assessments and hemiplegic neurologic tests and confirmed by computed tomography scanning and magnetic resonance imaging of the brain. The usual stay of hemiplegic stroke inpatients in the hospital rehabilitation unit was thirty-two days. The eligible 15 right and 15 left hemiplegic stroke patients without independent ambulatory ability were assigned to the intervention group, and the 15 right and 15 left hemiplegic stroke patients were selected as an age-matched, affected side-matched, and pre-walking-ability-matched control group that only received 4-week regular rehabilitation.

This current study (IRB number: CMUH103-REC2-034) was approved by the Research Ethics Committee, China Medical University Hospital and was conducted according to the Declaration of Helsinki. Sixty hemiplegic (30 right and 30 left) stroke patients pre-assessed without ambulatory ability met the inclusion criteria. All enrolled participants received experimental explanations and signed an informed consent form before their pre-assessment.

### 2.3. Non-Suspended Robot-Assisted Ambulatory Training

**Figure 1:**
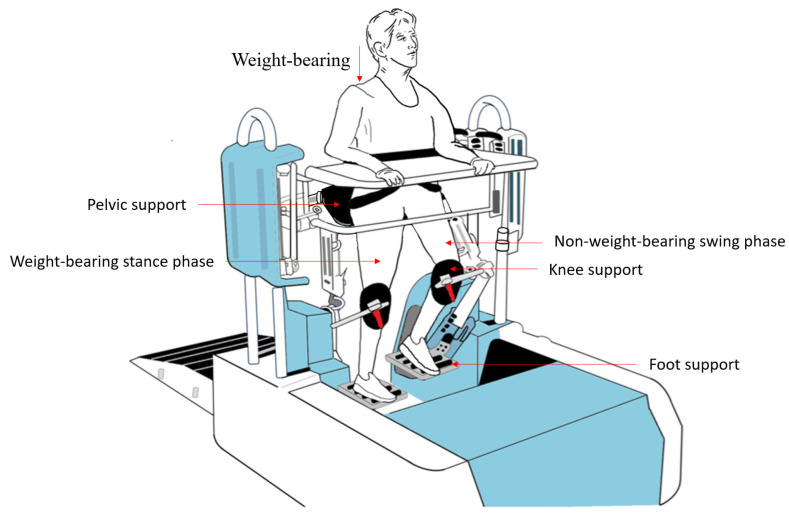
Non-suspended exoskeletal gait-training machine.

The HIWIN Robotic Gait-Training Device (Figure 1) (HIWIN Technologies Corp., Taichung, Taiwan) is a non-suspended robot-assisted ambulatory training machine (RAATM) designed to provide repetitive gait practice under near-normal physiological loading conditions (approximately 80–100% body-weight loading during the stance phase). The system incorporates a six-point support structure consisting of two knee supports, two foot supports, and anterior and posterior pelvic supports, enabling safe gait training without an overhead suspension system. Through its foot-based movable closed-kinetic-chain design, body weight is transmitted through the hips, femurs, knees, tibias, ankles, and feet during the stance phase, thereby facilitating physiological weight-bearing and task-specific gait training. The control algorithm coordinates reciprocal stepping movements between the affected and unaffected lower extremities. During the stance phase, approximately 80–100% physiological body-weight loading is transmitted through the lower extremities, whereas the swing limb is mechanically guided with minimal weight-bearing. Gait speed, step length, and step height can be adjusted according to individual patient tolerance and motor function. During the present study, training parameters were individually adjusted by licensed physical therapists to achieve safe, repetitive, and intensive gait practice. The robotic system was designed to assist rehabilitation practitioners in delivering thousands of repetitive ambulatory movements while reducing therapist workload and improving training consistency.

The non-suspended robot-assisted ambulatory training machine (RAATM) provides approximately 80–100% weight-bearing during the stance phase and 0% weight-bearing support during the swing phase.

### 2.4. Brunnstrom Stages

The Brunnstrom stages of stroke patients can help to understand progression after stroke: Brunnstrom Stage 1: Flaccidity; Brunnstrom Stage 2: Appearance of Spasticity; Brunnstrom Stage 3: Increased Spasticity; Brunnstrom Stage 4: Decreased Spasticity; Brunnstrom Stage 5: Complex Movement Combinations; and Brunnstrom Stage 6: Spasticity Disappears. Neurophysiological improvements in the lower extremities were graded on a scale of one to six according to the Brunnstrom stages by experienced physical therapists.

### 2.5. Balance Assessment

The four items of Balance Assessment in Sitting and Standing Positions are “static sitting balance”, “dynamic sitting balance”, “static standing balance”, and “dynamic standing balance”. The static sitting and standing balances were rated on 0–4 five-point scales, and the dynamic sitting and standing balances were rated on 0–3 four-point scales. The Balance Assessment, with total scores ranging from 0 to 14, was designed by experienced physical therapists to assess the stroke patients’ abilities to maintain static posture and perform dynamic tasks.

### 2.6. Barthel Index

The Barthel Index was measured by experienced occupational therapists and physical therapists to assess whether stroke patients can function and have independent mobility in their activities of daily living (ADL activities), i.e., feeding, bathing, grooming, dressing, bowel control, bladder control, toileting, chair transfer, ambulation and stair climbing.

### 2.7. Procedure

According to the inclusion and exclusion criteria, a total of 60 hemiplegic stroke patients (30 right-sided and 30 left-sided hemiplegia) without independent ambulatory ability at baseline were included in this study. All participants provided written informed consent prior to participation.

Thirty patients (15 right-sided and 15 left-sided hemiplegia) who received non-suspended robot-assisted ambulatory training in addition to conventional rehabilitation were included in the RAATM group (*n* = 30). The intervention was performed using the HIWIN Robotic Gait-Training System, which provided approximately 80–100% physiological weight-bearing during the stance phase and minimal weight-bearing during the swing phase. RAATM sessions were administered 3–5 days per week during hospitalization. Each session lasted approximately 15–20 min, resulting in a minimum accumulated training duration of 150 min (>4500 guided steps of the affected lower extremity) during the 4-week intervention period. The average accumulated training duration was 246 ± 74 min. Participants who received less than 150 min of robot-assisted ambulatory training during the study period were excluded from the RAATM group.

The control group consisted of 30 age-matched, affected-side-matched, and baseline ambulatory-status-matched stroke patients (15 right-sided and 15 left-sided hemiplegia) who received the same 4-week conventional rehabilitation program without robot-assisted training.

**Figure 2:**
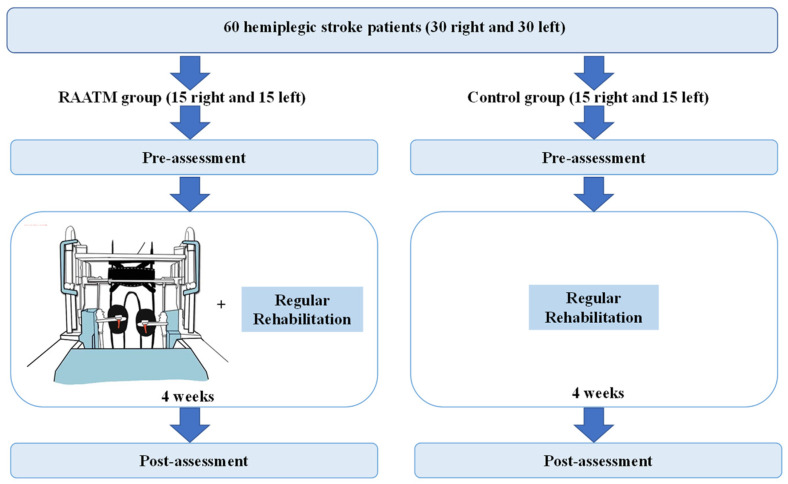
Flow chart. The RAATM group used a non-suspended robot-assisted ambulatory training machine with weight-bearing during the stance phase and without weight-bearing during the swing phase for longer than 150 min a month, plus a 4-week regular rehabilitation program. The age-matched, affect side-matched, pre-no-walking-ability-matched control group underwent a 4-week regular rehabilitation program only. Pre-assessment and post-assessment: Brunnstrom stages, Balance Assessment, and Barthel Index.

Baseline assessments included participant characteristics (age, hemiplegic side, language/swallowing impairment, cognitive impairment, and bladder dysfunction), Brunnstrom stages, Balance Assessment scores, and Barthel Index scores. The same outcome measures were re-assessed after completion of the 4-week intervention period. Changes in Brunnstrom stages, Balance Assessment scores, and Barthel Index scores were compared between the RAATM and control groups. A study flowchart is presented in Figure 2.

### 2.8. Statistical Analyses

All data are presented as mean ± standard deviation (SD). Data normality was assessed using the Kolmogorov–Smirnov test. Baseline demographic characteristics were compared between the RAATM and control groups using independent-samples Student’s *t*-tests. Within-group pre- and post-intervention comparisons were analyzed using paired-samples Student’s *t*-tests, whereas between-group differences in post-intervention scores and pre-to-post change scores (Post–Pre) were analyzed using independent-samples Student’s *t*-tests. Statistical analyses were performed using SPSS software version 22.0 (IBM SPSS Statistics, IBM Corp., Armonk, NY, USA). A two-tailed *p*-value < 0.05 was considered statistically significant. No missing data were identified among the enrolled participants; therefore, no imputation procedures were required.

## 3. Results

The characteristics of stroke patients in the control group versus the RAATM group are shown in Table 1. The number of stroke patients is 30 hemiplegia (15 left and 15 right) in the control group versus 30 hemiplegia (15 left and 15 right) in the RAATM group. The ages of the control group and RAATM group were similar.

The pre-assessment, post-assessment, and the difference from pre to post in the Brunnstrom stage of stroke patients in the control group versus the RAATM group are shown in Table 2. The pre-Brunnstrom stage in stroke patients was not different between the control group and the RAATM group. The post-Brunnstrom stage of the LE_Rt Score, post-Brunnstrom stage of the LE_Lt Score, (Post-Pre) Brunnstrom stage of the LE_Rt Score, and (Post-Pre) Brunnstrom stage of the LE_Lt Score in the RAATM group were significantly higher than those in the control group.

The pre-assessment, post-assessment, and the difference from pre to post in sitting balance and standing balance in stroke patients in the control group versus the RAATM group are shown in Table 3. Pre-sitting balance scores and pre-standing balance scores between the control group and the RAATM group were similar. The post-static sitting balance score, post-dynamic sitting balance score, post-static standing balance score, post-dynamic standing balance score, (Post-Pre) dynamic sitting balance score, (Post-Pre) static standing balance score, and (Post-Pre) dynamic standing balance score were significantly higher in the RAATM group than those in the control group (Table 3).

The pre-assessment, post-assessment, and the difference from pre to post in the Barthel Index for ADL activity in stroke patients in the control group versus the RAATM group are shown in Table 4. The pre-assessment of Barthel for ADL activity in stroke patients in the control group versus the RAATM group was similar. Post-mobility on level surfaces and post-stairs, (Post-Pre) mobility on level surfaces, (Post-Pre) stairs, and (Post-Pre) Barthel Total Score of the Barthel Index ADL Scale were significantly higher in the RAATM group than those in the control group.

## 4. Discussion

The present study demonstrated that non-suspended robot-assisted ambulatory training combined with conventional rehabilitation produced significantly greater improvements in lower-extremity motor recovery, balance performance, mobility ability, and activities of daily living than conventional rehabilitation alone in hemiplegic stroke patients who were unable to ambulate independently at baseline. Specifically, patients receiving more than 150 min of non-suspended robot-assisted ambulatory training (>4500 guided steps) during a 4-week intervention period showed significantly greater improvements in the Brunnstrom stage of the affected lower extremity, dynamic sitting balance, static standing balance, dynamic standing balance, mobility on level surfaces, stair-climbing ability, and total Barthel Index scores. These findings suggest that intensive non-suspended robotic gait training may facilitate functional recovery during the early post-stroke rehabilitation period. Notably, the scientific contribution of the present study lies not in the age of the dataset but in the unique rehabilitation strategy investigated. The majority of robot-assisted gait training studies have focused on suspension-based robotic systems that provide partial body-weight support during gait practice [7,9,10,12,14,19,20,22,29]. In contrast, the present robotic ambulatory training was specifically designed to provide approximately 80–100% physiological weight-bearing during the stance phase while maintaining mechanical guidance during the swing phase. To the best of our knowledge, no previous study has reported outcomes using this non-suspended physiological weight-bearing approach in stroke patients who lacked independent ambulatory ability at baseline. Consequently, the present study contributes novel evidence to an area that remains largely unexplored in contemporary stroke rehabilitation research.

The present findings are consistent with previous studies demonstrating beneficial effects of robot-assisted gait training on gait recovery and functional outcomes after stroke [6,13,19,22]. Ochi et al. [13] reported that a full-weight-bearing gait-assistance robot improved functional ambulation in non-ambulatory subacute stroke patients, while systematic reviews have concluded that robot-assisted gait training is particularly beneficial for patients with severe ambulatory impairment during the early stages of recovery [6,22]. Similarly, previous studies using Lokomat-assisted gait training and other robotic rehabilitation systems have reported improvements in balance, walking performance, and activities of daily living following stroke rehabilitation [9,19].

An important distinction between the present study and most previous investigations is the training strategy employed by the robotic system. Most commercially available robotic gait-training devices, including suspension-based exoskeletons and treadmill-assisted systems, utilize body-weight support mechanisms that partially unload the lower extremities during gait practice [7,10,12]. In contrast, the present robotic system was designed to provide approximately 80–100% physiological weight-bearing during the stance phase while mechanically guiding the swing phase under minimal weight-bearing conditions. Through its six-point support structure and closed-kinetic-chain design, the system enables repetitive gait practice under near-normal physiological loading conditions. To our knowledge, clinical evidence regarding non-suspended robot-assisted ambulatory training with near-normal physiological weight-bearing remains limited. Therefore, the present study extends the current literature by demonstrating that high-intensity non-suspended robotic gait training can improve lower-extremity motor recovery, balance, mobility, and functional independence in stroke patients with severe ambulatory impairment.

Several mechanisms may explain the observed improvements. First, repetitive gait practice under physiological weight-bearing conditions may enhance task-specific motor relearning through increased proprioceptive input and sensory feedback. Second, repeated symmetrical weight transfer during gait training may facilitate postural control and balance recovery. Previous studies have reported that repeated weight-bearing exercise improves both static and dynamic balance performance in stroke patients [30,31,32]. Third, the closed-kinetic-chain characteristics of the present robotic system may contribute to functional improvements. Closed-kinetic-chain exercises have been shown to improve balance and lower-extremity function more effectively than open-kinetic-chain exercises in some rehabilitation settings [33]. The combination of repetitive stepping practice, symmetrical weight-bearing, and near-normal loading conditions may therefore promote recovery of motor function and mobility after stroke.

Another clinically important finding is that the present intervention was effective in stroke patients who lacked independent ambulatory ability at baseline. Recovery after stroke is generally most rapid during the first several months following onset, and early intensive rehabilitation is considered critical for maximizing functional outcomes [5,29]. In the present study, patients with stroke onset within six months achieved significant improvements in motor recovery, balance, and mobility after only four weeks of intervention. These findings suggest that non-suspended robot-assisted ambulatory training may serve as a valuable adjunctive rehabilitation strategy during the subacute phase of stroke recovery, particularly for patients with severe gait impairment who require intensive and repetitive walking practice.

From a clinical perspective, the present robotic system may offer several practical advantages. The six-point support structure provides safety and stability during gait training while allowing near-normal weight-bearing through the lower extremities. In addition, robotic assistance enables the delivery of high-intensity, repetitive gait practice with consistent movement patterns, potentially reducing therapist workload while maintaining training quality. Collectively, the present findings support the integration of non-suspended robot-assisted ambulatory training into comprehensive stroke rehabilitation programs aimed at restoring walking ability and functional independence.

### Limitations

Several limitations should be acknowledged in this study. First, although the non-suspended robot-assisted ambulatory training was designed to provide approximately 80–100% physiological weight-bearing during the stance phase, biomechanical variables such as ankle dorsiflexion stiffness, joint kinematics, muscle activation patterns, and plantar pressure distribution were not evaluated. Previous studies have suggested that robot-assisted interventions may improve ankle function and lower-limb biomechanics after stroke [34]. Therefore, future studies should incorporate biomechanical assessments to better understand the mechanisms underlying functional recovery. Second, this study was conducted at a single rehabilitation center with a relatively limited sample size (*n* = 60). Although age-, affected-side-, and ambulatory-status-matched controls were used to minimize baseline differences, the findings may not be fully generalizable to broader stroke populations. Multi-center studies with larger sample sizes are warranted to confirm the reproducibility and external validity of the present findings. Third, the current study evaluated outcomes after a 4-week intervention period and did not include long-term follow-up assessments. Therefore, the durability of the observed improvements in lower-extremity motor function, balance, and activities of daily living remains unknown. Future studies should include follow-up evaluations at 3, 6, and 12 months after intervention to determine the long-term effectiveness of non-suspended robot-assisted ambulatory training. Finally, the present study employed a retrospective matched-control design rather than a randomized controlled trial. Although matching procedures were used to reduce potential confounding factors related to age, stroke severity, onset duration, and hemiplegic side, residual selection bias cannot be completely excluded. Future prospective randomized controlled trials stratified by stroke severity, post-stroke duration, age group, and hemiplegic side are needed to further validate the therapeutic effectiveness of this non-suspended robot-assisted ambulatory training.

## 5. Conclusions

This retrospective matched-control study demonstrated that non-suspended robot-assisted ambulatory training combined with conventional rehabilitation significantly improved lower-extremity motor recovery, standing balance, mobility performance, and activities of daily living in hemiplegic stroke patients who were unable to ambulate at baseline. Compared with matched controls receiving conventional rehabilitation alone, patients who received more than 150 min of robot-assisted ambulatory training (>4500 guided steps) during a 4-week intervention period exhibited significantly greater improvements in Brunnstrom stage of the lower extremities, static and dynamic standing balance, mobility on level surfaces, stair-climbing ability, and total Barthel Index scores.

A unique feature of the present robotic system is its non-suspended exoskeletal design, which enables approximately 80–100% physiological weight-bearing during the stance phase while mechanically guiding the swing phase. Unlike conventional suspension-based robotic gait trainers that partially unload the lower extremities, this approach provides a more realistic gait-training environment and promotes repetitive task-specific weight-bearing practice through a closed-kinetic-chain mechanism. The findings suggest that intensive non-suspended weight-bearing gait training may facilitate motor recovery and functional rehabilitation during the early post-stroke period in patients with severe ambulatory impairment. From a clinical perspective, the present system may reduce the physical demands placed on rehabilitation therapists while providing safe, high-intensity, and reproducible gait training for patients who are unable to walk independently. These characteristics make it a potentially valuable adjunct to conventional stroke rehabilitation programs. Future studies should validate these findings through prospective multi-center randomized controlled trials with larger sample sizes and longer follow-up periods. Additional investigations incorporating biomechanical assessments, such as ankle dorsiflexion stiffness, gait kinematics, muscle activation patterns, and plantar pressure distribution, may further clarify the mechanisms underlying functional recovery. The integration of emerging technologies, including virtual reality and non-invasive brain stimulation, may also enhance the therapeutic potential of non-suspended robot-assisted ambulatory training for post-stroke rehabilitation.

## Figures and Tables

**Table 1:** Characteristics of stroke patients in the control group versus the RAATM group.

Total number of stroke patients				*n* = 60					
		Control				RAATM		*p*	
Number of stroke patients		30				30			
Age, years old	61	±	14		58	±	10	NS	
Right hemiplegia		15				15			
Left hemiplegia		15				15			
Language/Swallowing Impairments		17				21			
Cognitive Impairments		6				3			
Bladder Function Impairments		6				6			
Duration of RAATM	0	±	0		246	±	74	*p* < 0.001	***

RAATM = robot-assisted ambulatory training system. Data = number of patients or mean ± standard deviation. NS = non-significant difference. *** Significant difference *p* < 0.001 between the control and RAATM groups (un-paired *t* test).

**Table 2:** Assessment of Brunnstrom stage in stroke patients in the control group versus the RAATM group.

		Control			RAATM		*p*	
Pre-Brunnstrom Stage UE-P-Rt	2.3	±	0.5	2.1	±	0.6	0.517	
Pre-Brunnstrom Stage UE-P-Lt	2.7	±	0.5	2.9	±	1.0	0.374	
Pre-Brunnstrom Stage UE-D-Rt	2.7	±	0.9	2.3	±	0.8	0.208	
Pre-Brunnstrom Stage UE-D-Lt	2.5	±	0.8	2.7	±	1.1	0.614	
Pre-Brunnstrom Stage LE-Rt	2.7	±	0.8	2.3	±	0.6	0.136	
Pre-Brunnstrom Stage LE-Lt	2.8	±	0.5	2.9	±	0.5	0.774	
Post-Brunnstrom Stage UE-P-Rt	2.6	±	0.6	2.4	±	0.8	0.463	
Post-Brunnstrom Stage UE-P-Lt	2.7	±	0.5	3.1	±	1.1	0.288	
Post-Brunnstrom Stage UE-D-Rt	2.7	±	1.0	2.4	±	0.8	0.318	
Post-Brunnstrom Stage UE-D-Lt	2.7	±	0.8	2.9	±	1.1	0.582	
Post-Brunnstrom Stage LE-Rt	3.1	±	1.1	4.1	±	0.6	0.004	**
Post-Brunnstrom Stage LE-Lt	3.0	±	0.6	3.8	±	0.7	0.002	**
(Post-Pre) Brunnstrom Stage UE-P-Rt	0.3	±	0.5	0.3	±	0.5	0.702	
(Post-Pre) Brunnstrom Stage UE-P-Lt	0.1	±	0.3	0.1	±	0.4	0.559	
(Post-Pre) Brunnstrom Stage UE-D-Rt	0.1	±	0.3	0.1	±	0.6	0.711	
(Post-Pre) Brunnstrom Stage UE-D-Lt	0.1	±	0.4	0.2	±	0.4	0.638	
(Post-Pre) Brunnstrom Stage LE-Rt	0.4	±	0.6	1.8	±	0.9	0.000	**
(Post-Pre) Brunnstrom Stage LE-Lt	0.2	±	0.4	0.9	±	0.8	0.004	**

RAATM = robot-assisted ambulatory training system. Brunnstrom Stage 1: Flaccidity; Brunnstrom Stage 2: Appearance of Spasticity; Brunnstrom Stage 3: Increased Spasticity; Brunnstrom Stage 4: Decreased Spasticity; Brunnstrom Stage 5: Complex Movement Combinations; Brunnstrom Stage 6: Spasticity Disappears. Data = mean ± standard deviation. Each group has *n* = 15. UE = upper extremity; P = proximal; Rt = right; Lt = left; LE = lower extremity; D = distal. ** Significant difference *p* < 0.01 between the control and RAATM groups (un-paired *t* test).

**Table 3:** Assessment of sitting balance and standing balance in stroke patients in the control group versus the RAATM group.

		Control			RAATM		*p*	
Pre-Static Sitting Balance Score	2.2	±	0.7	2.4	±	0.7	0.279	
Pre-Dynamic Sitting Balance Score	1.6	±	0.6	1.7	±	0.5	0.661	
Pre-Static Standing Balance Score	1.0	±	0.6	1.2	±	0.6	0.174	
Pre-Dynamic Standing Balance Score	0.9	±	0.3	0.9	±	0.3	0.398	
Post-Static Sitting Balance Score	2.5	±	0.7	2.9	±	0.3	0.006	**
Post-Dynamic Sitting Balance Score	1.8	±	0.6	2.3	±	0.6	0.005	**
Post-Static Standing Balance Score	1.2	±	0.6	1.7	±	0.6	0.003	**
Post-Dynamic Standing Balance Score	1.1	±	0.4	1.6	±	0.6	0.0003	***
(Post-Pre) Static Sitting Balance Score	0.4	±	0.6	0.6	±	0.7	0.325	
(Post-Pre) Dynamic Sitting Balance Score	0.2	±	0.4	0.6	±	0.6	0.010	*
(Post-Pre) Static Standing Balance Score	0.2	±	0.4	0.5	±	0.5	0.014	*
(Post-Pre) Dynamic Standing Balance Score	0.2	±	0.4	0.7	±	0.8	0.004	**

RAATM = robot-assisted ambulatory training system. The static sitting and standing balances were rated on 0–4 five-point scales, and the dynamic sitting and standing balances were rated on 0–3 four-point scales. Data = mean ± standard deviation. Each group *n* = 30. * Significant difference *p* < 0.05, ** significant difference *p* < 0.01, and *** significant difference *p* < 0.001 between the control and RAATM groups (un-paired *t* test).

**Table 4:** Assessment of the Barthel for activities of daily living (ADL) scale in stroke patients in the control group versus the RAATM group.

		Control			RAATM		*p*	
Pre-Feeding (10,5,0)	3.3	±	3.8	3.7	±	3.7	0.732	
Pre-Grooming (5,0)	1.2	±	2.2	1.5	±	2.3	0.567	
Pre-Bowel control (10,5,0)	4.2	±	3.5	4.0	±	3.1	0.845	
Pre-Bladder control (10,5,0)	4.3	±	3.1	4.5	±	3.0	0.835	
Pre-Dressing (10,5,0)	2.8	±	2.5	2.3	±	2.5	0.447	
Pre-Transfers (bed to chair) (15,10,5,0)	4.5	±	2.4	4.2	±	3.0	0.634	
Pre-Toilet use (10,5,0)	2.3	±	2.5	1.8	±	2.5	0.441	
Pre-Mobility on level surfaces (15,10,5,0)	0			0				
Pre-Stairs (10,5,0)	0			0				
Pre-Bathing (5,0)	0			0				
Pre-Barthel Total Score	22.7	±	13.2	22.0	±	14.0	0.850	
Post-Feeding (10,5,0)	4.0	±	3.8	4.7	±	4.1	0.519	
Post-Grooming (5,0)	1.5	±	2.3	2.0	±	2.5	0.425	
Post-Bowel control (10,5,0)	5.0	±	3.7	5.7	±	3.9	0.499	
Post-Bladder control (10,5,0)	4.8	±	3.6	5.7	±	3.4	0.360	
Post-Dressing (10,5,0)	3.2	±	2.5	2.8	±	2.5	0.605	
Post-Transfers (bed to chair) (15,10,5,0)	6.7	±	3.6	7.7	±	2.9	0.235	
Post-Toilet use (10,5,0)	2.7	±	2.5	2.5	±	2.5	0.800	
Post-Mobility on level surfaces (15,10,5,0)	2.7	±	2.5	4.2	±	3.0	0.039	*
Post-Stairs (10,5,0)	0			0.7	±	1.7	0.039	*
Post-Bathing (5,0)	0			0				
Post-Barthel Total Score	30.5	±	17.1	35.8	±	15.3	0.208	
(Post-Pre) Feeding (10,5,0)	0.7	±	1.7	1.0	±	2.8	0.577	
(Post-Pre) Grooming (5,0)	0.3	±	1.3	0.5	±	1.5	0.647	
(Post-Pre) Bowel control (10,5,0)	0.8	±	1.9	1.7	±	2.7	0.175	
(Post-Pre) Bladder control (10,5,0)	0.5	±	1.5	1.2	±	2.2	0.171	
(Post-Pre) Dressing (10,5,0)	0.3	±	1.3	0.5	±	1.5	0.647	
(Post-Pre) Transfers bed to chair (15,10,5,0)	2.2	±	2.5	3.5	±	3.7	0.111	
(Post-Pre) Toilet use (10,5,0)	0.3	±	1.8	0.7	±	2.2	0.522	
(Post-Pre) Mobility on level surfaces (15,10,5,0)	2.7	±	2.5	4.2	±	3.0	0.039	*
(Post-Pre) Stairs (10,5,0)	0			0.7	±	1.7	0.039	*
(Post-Pre) Bathing (5,0)	0			0				
(Post-Pre) Barthel Total Score	7.8	±	6.0	13.8	±	9.2	0.004	**

RAATM = robot-assisted ambulatory training system. Data = mean ± standard deviation. Each group has *n* = 30. * Significant difference *p* < 0.05 and ** significant difference *p* < 0.01 between the control and RAATM groups (un-paired *t* test).

## Data Availability

The raw data supporting the conclusions of this article will be made available by the authors on request.
